# Supplementary material for: Molecular characterization of the sea lamprey retina illuminates the evolutionary origin of retinal cell types
Source: Nat Commun. 2024 Dec 30;15:10761. doi: 10.1038/s41467-024-55019-x (PMC11685597; doi:10.1038/s41467-024-55019-x)
Supplement: Supplementary file 1 — Supplementary Information [file 41467_2024_55019_MOESM1_ESM.pdf]

Supplementary Information

Supplementary Figure 1-11

Supplementary Table 1-5

Figure S1

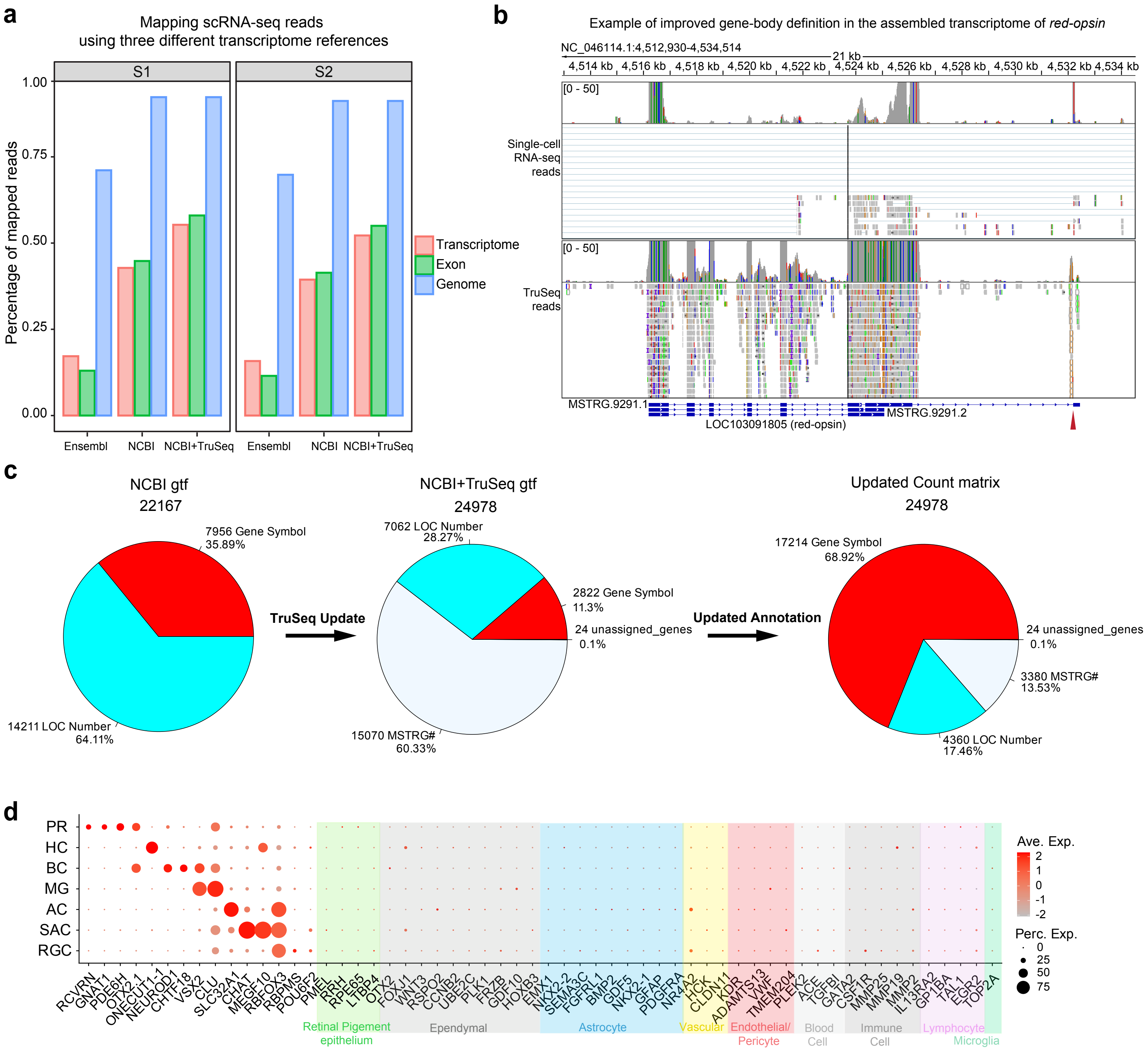
a
Mapping scRNA-seq reads
using three different transcriptome references
S1
S2
Percentage of mapped reads
1.00
0.75
0.50
0.25
0.00
Ensembl
NCBI
NCBI+TruSeq
Transcriptome
Exon
Genome
b
Example of improved gene-body definition in the assembled transcriptome of red-opsin
NC_046114.1:4,512,930-4,534,514
21 kb
4,514 kb
4,516 kb
4,518 kb
4,520 kb
4,522 kb
4,524 kb
4,526 kb
4,528 kb
4,530 kb
4,532 kb
4,534 kb
[0 - 50]
Single-cell
RNA-seq
reads
TruSeq
reads
MSTRG.9291.1
MSTRG.9291.2
LOC103091805 (red-opsin)
c
NCBI gtf
22167
7956 Gene Symbol
35.89%
14211 LOC Number
64.11%
TruSeq Update
NCBI+TruSeq gtf
24978
7062 LOC Number
28.27%
2822 Gene Symbol
11.3%
24 unassigned_genes
0.1%
15070 MSTRG#
60.33%
Updated Annotation
Updated Count matrix
24978
17214 Gene Symbol
68.92%
24 unassigned_genes
0.1%
3380 MSTRG#
13.53%
4360 LOC Number
17.46%
d
PR
HC
BC
MG
AC
SAC
RGC
RCVRN
GNAT1
PDE6H
OTX2.1
ONECUT1-1
NEUROD1
CHTF18
VSX2
CLU
SLC32A1
CHAT
MEGF10
RBFOX3
RBPMS
POU6F2
PMEL
RRH
RPE65
LTBP4
OTX2
FOXJ1
WNT3
RSPO2
CCNB2
UBE2C
PLK1
FRZB
GDF10
HOXB3
EMX1
NKX2-2
SEMA3C
FGFRL1
BMP2
GDF5
NKX2-1
GFAP
PDGFRA
NR4A2
HCK
CLDN11
KDR
ADAMTS13
VWF
TMEM204
PLEK2
ACE
TGFBI
GATA2
CSF1R
MMP25
MMP19
MMP2
IL13RA2
GP1BA
TAL1
EGR2
TOP2A
Retinal Pigment
epithelium
Ependymal
Astrocyte
Vascular
Endothelial/
Pericyte
Blood
Cell
Immune
Cell
Lymphocyte
Microglia
Ave. Exp.
2
1
0
-1
-2
Perc. Exp.
0
25
50
75

Supplementary Figure 1. Assembly and annotation of the retina-specific transcriptome.
(a) Bar plots showing mapping percentages of scRNA-seq reads to “Transcriptome,” “Exon,” and “Genome” from two biological replicates (S1 and S2) with Ensembl (Pmarinus_7.0), NCBI (kPetMar1.pri), or the updated NCBI+TruSeq transcriptome reference. See Source Data.
(b) Improved gene-body definition for the *red-opsin* gene in the NCBI+TruSeq transcriptome. The alignment of scRNA-seq (top panel) and TruSeq (bottom panel) reads to the “*red-opsin”* locus was visualized with the Integrated Genomics Viewer (IGV). Red arrowhead indicates a newly identified exon region of the *red-opsin* gene.
(c) Pie charts showing the proportions of genes annotated with LOC numbers, MSTRG numbers, or gene symbols in the raw count matrices. Three different gtf files are compared: the NCBI gtf, the NCBI+TruSeq gtf, and the NCBI+TruSeq gtf with updated gene annotation. The NCBI+TruSeq gtf file contains more genes than the NCBI gtf file due to the inclusion of newly identified transcripts following the TruSeq update. Most unassigned genes are mitochondrial tRNAs. See Source Data.
(d) Dot plot showing the expression patterns of canonical markers for retinal cell classes and non-neuronal cell classes, such as retinal pigmented epithelium, ependymal cells, astrocyte, etc. See Source Data.

Figure S2

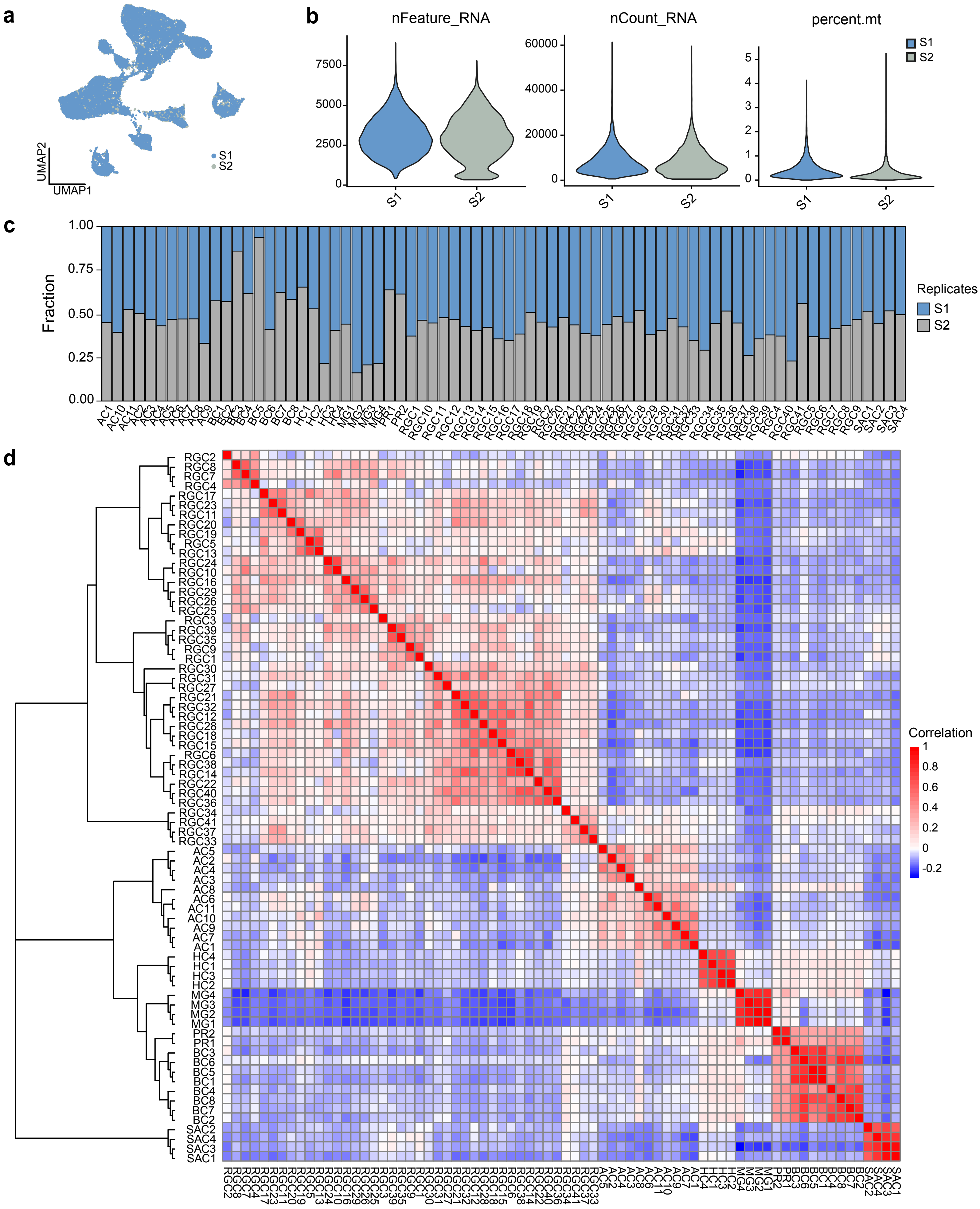

a
UMAP2
UMAP1
S1
S2
b
nFeature_RNA
nCount_RNA
percent.mt
S1
S2
c
Fraction
Replicates
S1
S2
d
Correlation

Supplementary Figure 2. Quality metrics of lamprey scRNA-seq data and cell type hierarchical relationships.

(a) UMAP visualization of all lamprey cells, as depicted in Figure 1c, but here colored by distinct replicates to demonstrate lack of bias associated with different replicates.

(b) Violin plots showing distributions of the number of expressed genes (nFeature_RNA), RNA counts, and percentages of mitochondrial genes (percent.mt) detected in each replicate.

(c) Bar plot displaying fractions of cells from each replicate across individual cell types. See Source Data.

(d) Hierarchical clustering and heatmap showing the Pearson correlation coefficients calculated for each pair of cell types, using the top 3,000 highly variable genes. See Source Data.

Figure S3

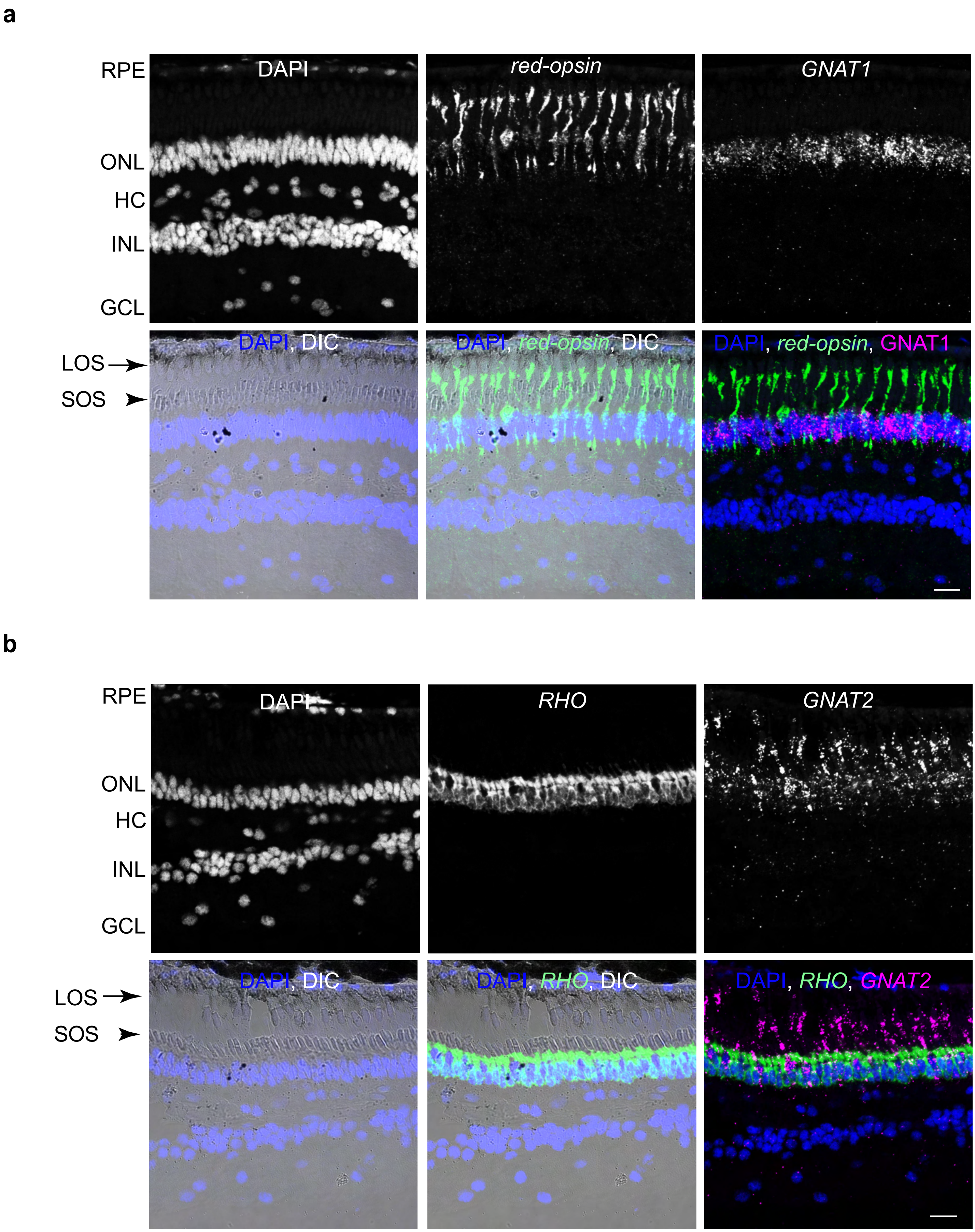
a
RPE
ONL
HC
INL
GCL
LOS
SOS
DAPI
red-opsin
GNAT1
DAPI, DIC
DAPI, red-opsin, DIC
DAPI, red-opsin, GNAT1
b
RPE
ONL
HC
INL
GCL
LOS
SOS
DAPI
RHO
GNAT2
DAPI, DIC
DAPI, RHO, DIC
DAPI, RHO, GNAT2

Supplementary Figure 3. Fluorescence in situ validation of marker genes for lamprey PR types

(a) Fluorescence in situ hybridization (FISH) validations confirming exclusive expression patterns of *red-opsin* and *GNAT1* in PR2 and PR1. *Red-opsin* (green in the merged image) is expressed by PRs with long outer segments (LOS, indicated by the arrow); while *GNAT1* (magenta in the merged image) is expressed by PRs with short outer segments (SOS, indicated by the arrowhead). The LOS and SOS structures are more clearly visualized in the differential interference contrast (DIC) image.

(b) FISH validation showing the exclusive expression of *rhodopsin* (*RHO*) and *GNAT2* in PR1 and PR2. *RHO* (green in the merged image) is expressed by PRs with short outer segments (SOS, arrowhead); while *GNAT2* (magenta in the merged image) is expressed by PRs with long outer segments (LOS, arrow).

Nuclei stained with DAPI are in blue. Scale bar, 20 μm.

Figure S4

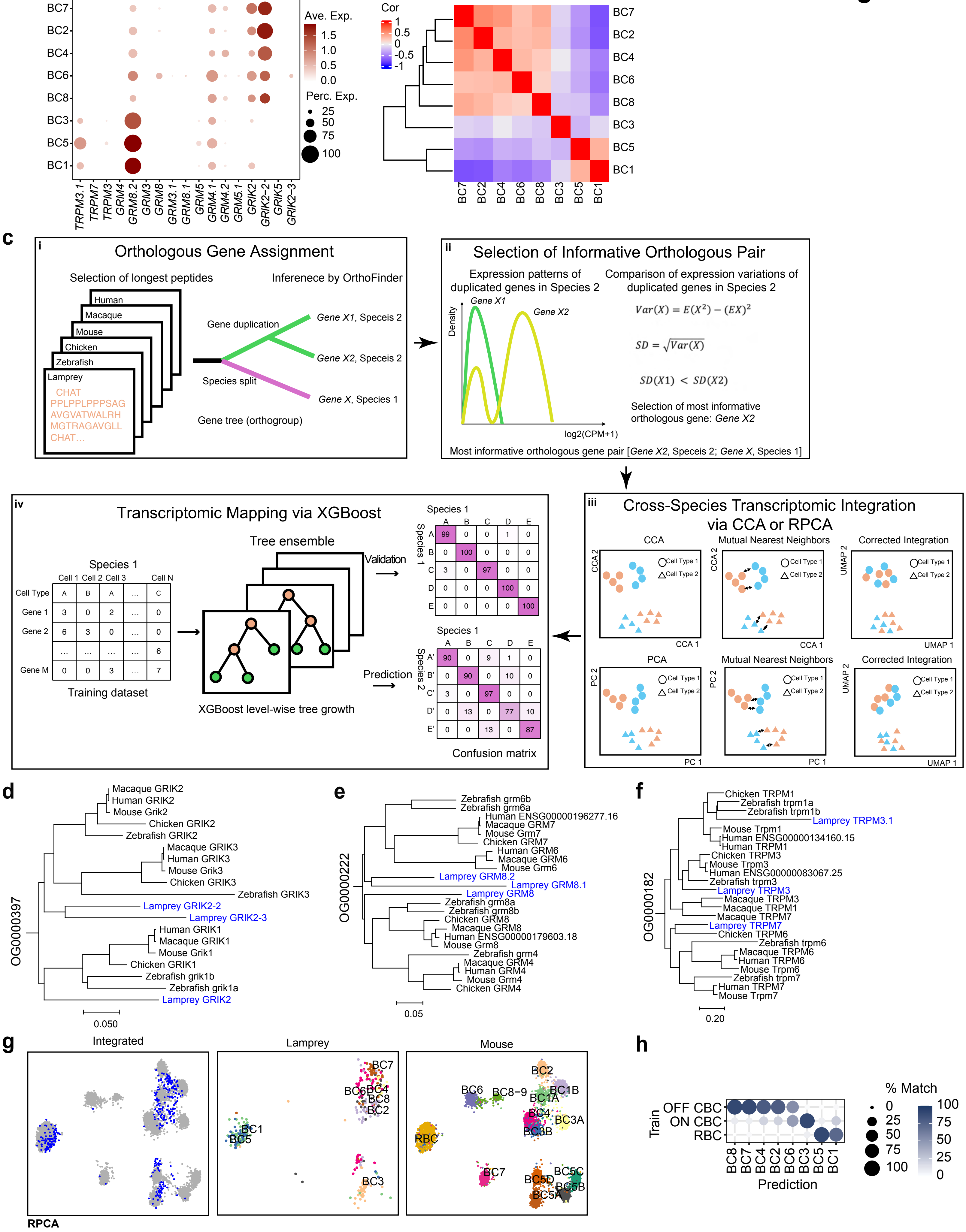

a
BC7
BC2
BC4
BC6
BC8
BC3
BC5
BC1
Ave. Exp.
1.5
1.0
0.5
0.0
Perc. Exp.
25
50
75
100
TRPM3.1
TRPM7
TRPM3
GRM4
GRM8.2
GRM3
GRM8
GRM3.1
GRM8.1
GRM5
GRM4.1
GRM4.2
GRM5.1
GRIK2
GRIK2-2
GRIK5
GRIK2-3
b
Cor
1
0.5
0
-0.5
-1
c
i
Orthologous Gene Assignment
Selection of longest peptides
Inferenece by OrthoFinder
Human
Macaque
Mouse
Chicken
Zebrafish
Lamprey
CHAT
PPLPPLPPPSAG
AVGVATWALRH
MGTRAGAVGLL
CHAT…
Gene duplication
Species split
Gene X1, Speceis 2
Gene X2, Speceis 2
Gene X, Species 1
Gene tree (orthogroup)
ii
Selection of Informative Orthologous Pair
Expression patterns of duplicated genes in Species 2
Comparison of expression variations of duplicated genes in Species 2
Gene X1
Gene X2
Density
log2(CPM+1)
$Var(X) = E(X^2) - (EX)^2$
$SD = \sqrt{Var(X)}$
$SD(X1) < SD(X2)$
Selection of most informative orthologous gene: Gene X2
Most informative orthologous gene pair [Gene X2, Speceis 2; Gene X, Species 1]
iii
Cross-Species Transcriptomic Integration via CCA or RPCA
CCA
Mutual Nearest Neighbors
Corrected Integration
PCA
Cell Type 1
Cell Type 2
CCA 1
CCA 2
PC 1
PC 2
UMAP 1
UMAP 2
iv
Transcriptomic Mapping via XGBoost
Species 1
Cell 1 Cell 2 Cell 3 Cell N
Cell Type
Gene 1
Gene 2
Gene M
Training dataset
Tree ensemble
XGBoost level-wise tree growth
Validation
Prediction
Species 2
Confusion matrix
d
OG0000397
Macaque GRIK2
Human GRIK2
Mouse Grik2
Chicken GRIK2
Zebrafish GRIK2
Macaque GRIK3
Human GRIK3
Mouse Grik3
Chicken GRIK3
Zebrafish GRIK3
Lamprey GRIK2-2
Lamprey GRIK2-3
Human GRIK1
Macaque GRIK1
Mouse Grik1
Chicken GRIK1
Zebrafish grik1b
Zebrafish grik1a
Lamprey GRIK2
0.050
e
OG0000222
Zebrafish grm6b
Zebrafish grm6a
Human ENSG00000196277.16
Macaque GRM7
Mouse Grm7
Chicken GRM7
Human GRM6
Macaque GRM6
Mouse Grm6
Lamprey GRM8.2
Lamprey GRM8.1
Lamprey GRM8
Zebrafish grm8a
Zebrafish grm8b
Chicken GRM8
Macaque GRM8
Human ENSG00000179603.18
Mouse Grm8
Zebrafish grm4
Macaque GRM4
Human GRM4
Mouse Grm4
Chicken GRM4
0.05
f
OG0000182
Chicken TRPM1
Zebrafish trpm1a
Zebrafish trpm1b
Lamprey TRPM3.1
Mouse Trpm1
Human ENSG00000134160.15
Human TRPM1
Chicken TRPM3
Mouse Trpm3
Human ENSG00000083067.25
Zebrafish trpm3
Lamprey TRPM3
Macaque TRPM3
Macaque TRPM1
Macaque TRPM7
Lamprey TRPM7
Chicken TRPM6
Zebrafish trpm6
Macaque TRPM6
Human TRPM6
Mouse Trpm6
Zebrafish trpm7
Human TRPM7
Mouse Trpm7
0.20
g
Integrated
Lamprey
Mouse
RPCA
h
% Match
Train
OFF CBC
ON CBC
RBC
Prediction

Supplementary Figure 4. Gene expression in lamprey BC types and cross-species integration of BC types.
(a) Dot plot showing the expression patterns of genes from the *TRPM*, *GRM*, and *GRIK* families across lamprey BC types. See Source Data.
(b) Hierarchical clustering and heatmap displaying Pearson correlation coefficients calculated between each pair of BC types using the top 3000 highly variable genes. The dendrogram on the left shows their hierarchical relationships, constructed from agglomerative hierarchical clustering based on correlation distance. See Source Data.
(c) The analysis workflow for cross-species comparison of cell types. i) Identification of orthologous relationships of genes across lamprey, zebrafish, chicken, mouse, macaque, and human via OrthoFinder. ii) Identification of the most informative orthologous genes when multiple orthologous genes are present in the dataset. iii) Transcriptomic integration via CCA or RPCA. iv) Transcriptomic mapping via XGBoost.
(d) Phylogenetic tree of ionotropic glutamate receptor genes among the selected five species, inferred from OrthoFinder. Scale bar refers to a phylogenetic distance of 0.05 substitutions per site. The orthogroup name is shown on the left.
(e) Phylogenetic tree of metabolic glutamate receptor genes among the five species.
(f) Phylogenetic tree of transient receptor potential TRPM genes among the five species.
(g) Transcriptomic integration of lamprey and mouse BC types via RPCA.
(h) Confusion matrix of the correspondence of lamprey BC types to mouse BC subclasses. Mouse BC subclasses were used as the training dataset. See Source Data.

Figure S5

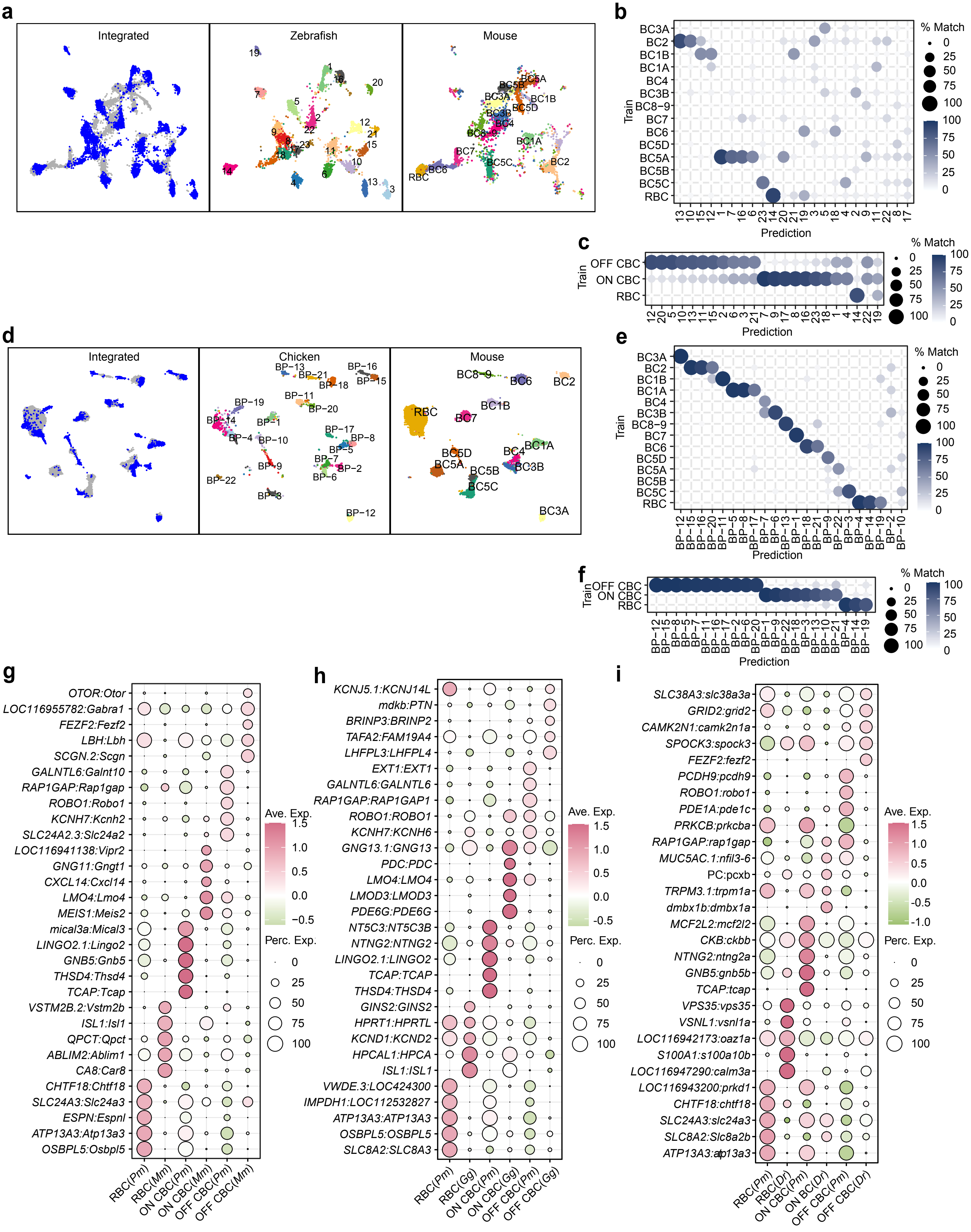

a
Integrated
Zebrafish
Mouse
b
Train
Prediction
% Match
c
OFF CBC
ON CBC
RBC
d
Integrated
Chicken
Mouse
e
f
g
OTOR:Otor
LOC116955782:Gabra1
FEZF2:Fezf2
LBH:Lbh
SCGN.2:Scgn
GALNTL6:Galntl10
RAP1GAP:Rap1gap
ROBO1:Robo1
KCNH7:Kcnh2
SLC24A2.3:Slc24a2
LOC116941138:Vipr2
GNG11:Gngt1
CXCL14:Cxcl14
LMO4:Lmo4
MEIS1:Meis2
mical3a:Mical3
LINGO2.1:Lingo2
GNB5:Gnb5
THSD4:Thsd4
TCAP:Tcap
VSTM2B.2:Vstm2b
ISL1:Isl1
QPCT:Qpct
ABLIM2:Ablim1
CA8:Car8
CHTF18:Chtf18
SLC24A3:Slc24a3
ESPN:Espnl
ATP13A3:Atp13a3
OSBPL5:Osbpl5
RBC(Pm)
RBC(Mm)
ON CBC(Pm)
ON CBC(Mm)
OFF CBC(Pm)
OFF CBC(Mm)
Ave. Exp.
Perc. Exp.
h
KCNJ5.1:KCNJ14L
mdkb:PTN
BRINP3:BRINP2
TAFA2:FAM19A4
LHFPL3:LHFPL4
EXT1:EXT1
GALNTL6:GALNTL6
RAP1GAP:RAP1GAP1
ROBO1:ROBO1
KCNH7:KCNH6
GNG13.1:GNG13
PDC:PDC
LMO4:LMO4
LMOD3:LMOD3
PDE6G:PDE6G
NT5C3:NT5C3B
NTNG2:NTNG2
LINGO2.1:LINGO2
TCAP:TCAP
THSD4:THSD4
GINS2:GINS2
HPRT1:HPRTL
KCND1:KCND2
HPCAL1:HPCA
ISL1:ISL1
VWDE.3:LOC424300
IMPDH1:LOC112532827
ATP13A3:ATP13A3
OSBPL5:OSBPL5
SLC8A2:SLC8A3
RBC(Pm)
RBC(Gg)
ON CBC(Pm)
ON CBC(Gg)
OFF CBC(Pm)
OFF CBC(Gg)
i
SLC38A3:slc38a3a
GRID2:grid2
CAMK2N1:camk2n1a
SPOCK3:spock3
FEZF2:fezf2
PCDH9:pcdh9
ROBO1:robo1
PDE1A:pde1c
PRKCB:prkcba
RAP1GAP:rap1gap
MUC5AC.1:nfil3-6
PC:pcxb
TRPM3.1:trpm1a
dmbx1b:dmbx1a
MCF2L2:mcf2l2
CKB:ckbb
NTNG2:ntng2a
GNB5:gnb5b
TCAP:tcap
VPS35:vps35
VSNL1:vsnl1a
LOC116942173:oaz1a
S100A1:s100a10b
LOC116947290:calm3a
LOC116943200:prkd1
CHTF18:chtf18
SLC24A3:slc24a3
SLC8A2:Slc8a2b
ATP13A3:atp13a3
RBC(Pm)
RBC(Dr)
ON CBC(Pm)
ON BC(Dr)
OFF CBC(Pm)
OFF CBC(Dr)

Supplementary Figure 5. Cross-species comparison of BC types.
(a) Transcriptomic integration of zebrafish and mouse BC types via RPCA.
(b) Confusion matrix of the correspondence of zebrafish BC types to mouse BC types. Mouse BC types were used as a training dataset. See Source Data.
(c) Confusion matrix of the correspondence of zebrafish BC types to mouse BC subclasses. Mouse BC subclasses were used as a training dataset. See Source Data.
(d) Transcriptomic integration of chicken and mouse BC types via RPCA.
(e) Confusion matrix of the correspondence of chicken BC types to mouse BC types. Mouse BC types were used as a training dataset. See Source Data.
(f) Confusion matrix of the correspondence of chicken BC types to mouse BC subclasses. Mouse BC subclasses were used as a training dataset. See Source Data.
(g-i) Dot plots showing species-specific markers of BC subclasses between lamprey and mouse (g), lamprey and chicken (h), and lamprey and zebrafish (i). See Source Data.

Figure S6

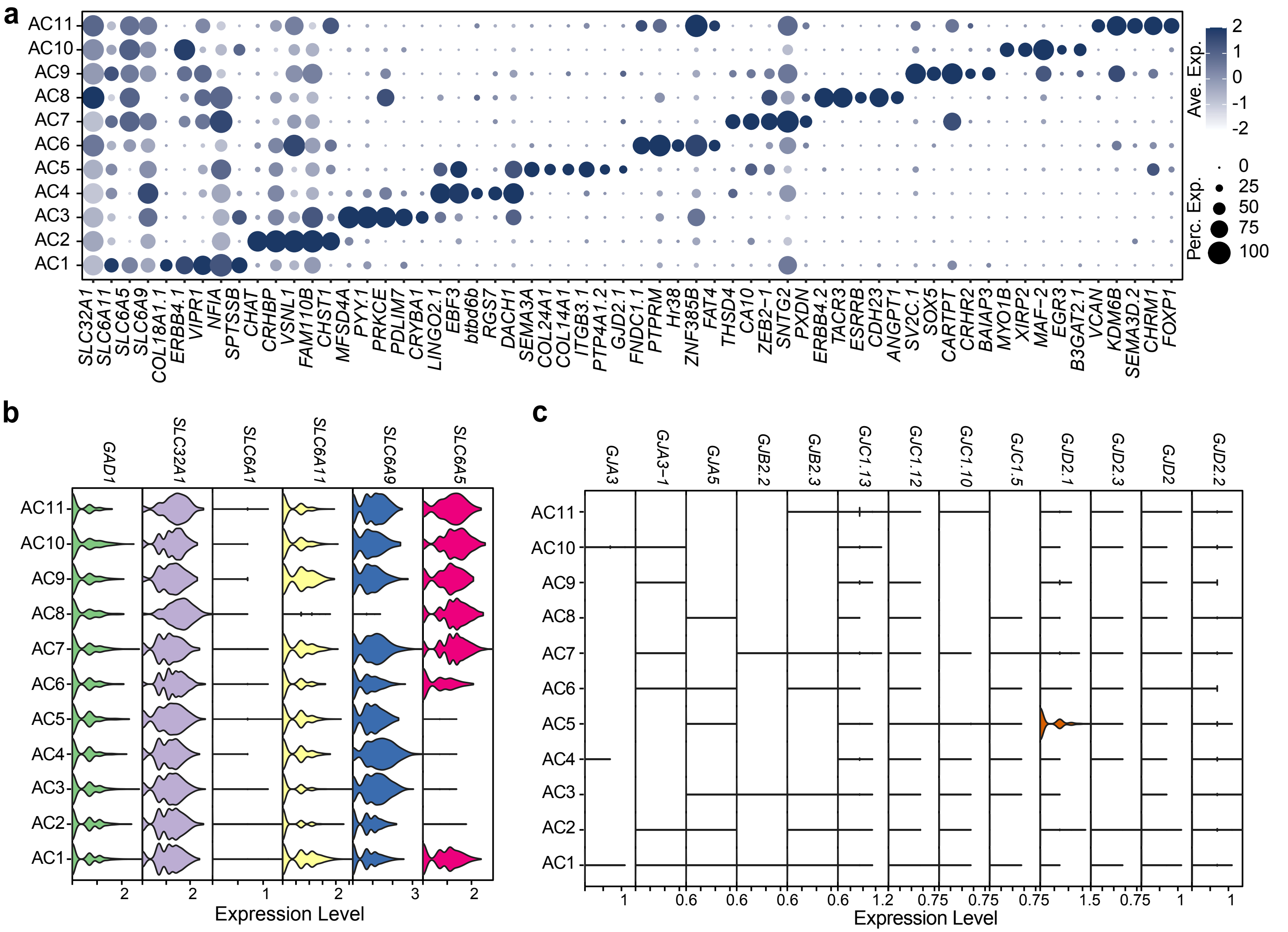

a
AC11
AC10
AC9
AC8
AC7
AC6
AC5
AC4
AC3
AC2
AC1
SLC32A1
SLC6A11
SLC6A5
SLC6A9
COL18A1.1
ERBB4.1
VIPR1
NFIA
SPTSSB
CHAT
CRHBP
VSNL1
FAM110B
CHST1
MFSD4A
PYY.1
PRKCE
PDLIM7
CRYBA1
LINGO2.1
EBF3
btbd6b
RGS7
DACH1
SEMA3A
COL24A1
COL14A1
ITGB3.1
PTP4A1.2
GJD2.1
FNDC1.1
PTPRM
Hr38
ZNF385B
FAT4
THSD4
CA10
ZEB2−1
SNTG2
PXDN
ERBB4.2
TACR3
ESRRB
CDH23
ANGPT1
SV2C.1
SOX5
CARTPT
CRHR2
BAIAP3
MYO1B
XIRP2
MAF−2
EGR3
B3GAT2.1
VCAN
KDM6B
SEMA3D.2
CHRM1
FOXP1
Ave. Exp.
2
1
0
-1
-2
Perc. Exp.
0
25
50
75
100
b
GAD1
SLC32A1
SLC6A1
SLC6A11
SLC6A9
SLC6A5
Expression Level
c
GJA3
GJA3−1
GJA5
GJB2.2
GJB2.3
GJC1.13
GJC1.12
GJC1.10
GJC1.5
GJD2.1
GJD2.3
GJD2
GJD2.2
Expression Level

Supplementary Figure 6. Gene expression in lamprey AC types

(a) Dot plot showing the expression of genes for GABA transporters (*SLC32A1* and *SLC6A11*) and glycine transporters (*SLC6A5* and *SLC6A9*) together with marker genes for individual AC clusters. See Source Data.

(b) Stacked violin plot showing the expression patterns of all GABA and glycine transporters in lamprey AC types.

(c) Stacked violin plot showing the expression patterns of all gap junction genes in lamprey AC types.

Figure S7

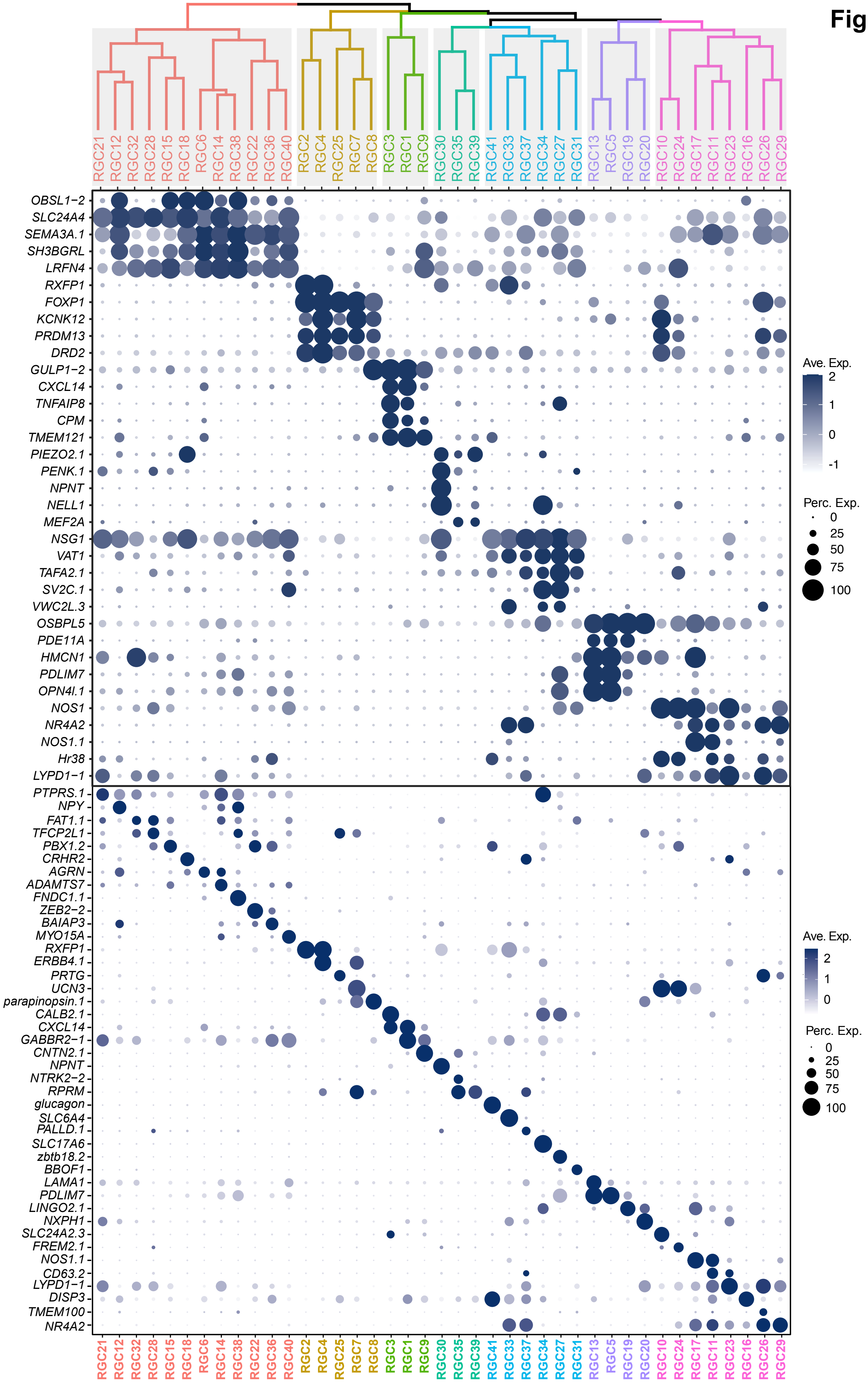

RGC21 RGC12 RGC32 RGC28 RGC15 RGC18 RGC6 RGC14 RGC38 RGC22 RGC36 RGC40 RGC2 RGC4 RGC25 RGC7 RGC8 RGC3 RGC1 RGC9 RGC30 RGC35 RGC39 RGC41 RGC33 RGC37 RGC34 RGC27 RGC31 RGC13 RGC5 RGC19 RGC20 RGC10 RGC24 RGC17 RGC11 RGC23 RGC16 RGC26 RGC29
OBSL1−2
SLC24A4
SEMA3A.1
SH3BGRL
LRFN4
RXFP1
FOXP1
KCNK12
PRDM13
DRD2
GULP1−2
CXCL14
TNFAIP8
CPM
TMEM121
PIEZO2.1
PENK.1
NPNT
NELL1
MEF2A
NSG1
VAT1
TAFA2.1
SV2C.1
VWC2L.3
OSBPL5
PDE11A
HMCN1
PDLIM7
OPN4l.1
NOS1
NR4A2
NOS1.1
Hr38
LYPD1−1
Ave. Exp.
2
1
0
-1
Perc. Exp.
0
25
50
75
100
PTPRS.1
NPY
FAT1.1
TFCP2L1
PBX1.2
CRHR2
AGRN
ADAMTS7
FNDC1.1
ZEB2−2
BAIAP3
MYO15A
RXFP1
ERBB4.1
PRTG
UCN3
parapinopsin.1
CALB2.1
CXCL14
GABBR2−1
CNTN2.1
NPNT
NTRK2−2
RPRM
glucagon
SLC6A4
PALLD.1
SLC17A6
zbtb18.2
BBOF1
LAMA1
PDLIM7
LINGO2.1
NXPH1
SLC24A2.3
FREM2.1
NOS1.1
CD63.2
LYPD1−1
DISP3
TMEM100
NR4A2
Ave. Exp.
2
1
0
Perc. Exp.
0
25
50
75
100
RGC21 RGC12 RGC32 RGC28 RGC15 RGC18 RGC6 RGC14 RGC38 RGC22 RGC36 RGC40 RGC2 RGC4 RGC25 RGC7 RGC8 RGC3 RGC1 RGC9 RGC30 RGC35 RGC39 RGC41 RGC33 RGC37 RGC34 RGC27 RGC31 RGC13 RGC5 RGC19 RGC20 RGC10 RGC24 RGC17 RGC11 RGC23 RGC16 RGC26 RGC29

Supplementary Figure 7. Expression patterns of marker genes for RGC subgroups and types.

The dendrogram at the top shows the hierarchical relationships among RGC types, constructed from agglomerative hierarchical clustering based on correlation distance. RGC types are grouped into seven subgroups based on Pearson correlation coefficients calculated between each pair of RGC types from the top 3,000 highly variable genes. These seven RGC subgroups are visually distinguished by different colors and enclosed within shadow boxes. Subgroup-specific markers are shown in the top dot plot, while the expression patterns of markers for individual RGC types are presented in the bottom dot plot. See Source Data.

Figure S8

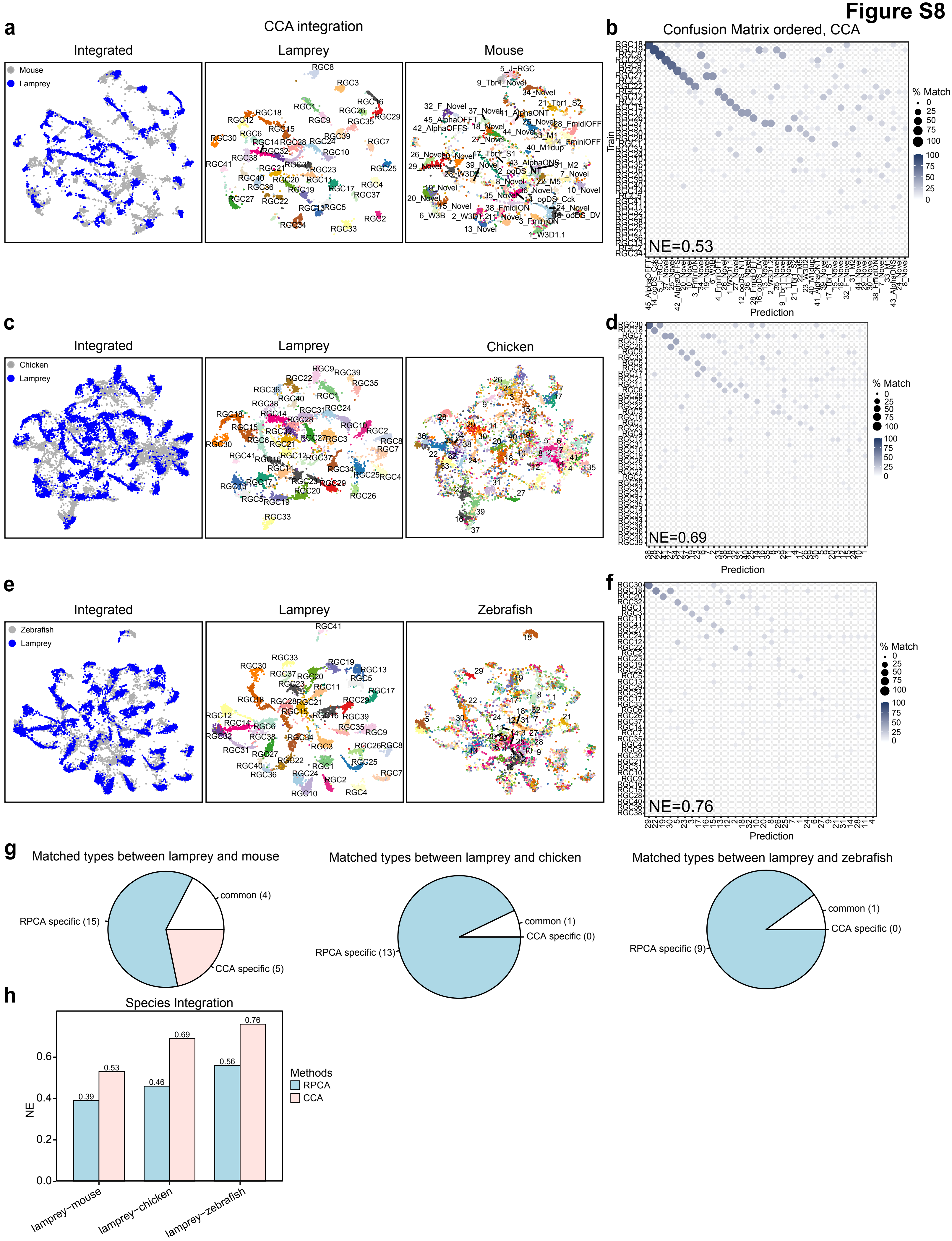
a
CCA integration
Integrated
Lamprey
Mouse
b
Confusion Matrix ordered, CCA
NE=0.53
Train
Prediction
% Match
c
Integrated
Lamprey
Chicken
d
NE=0.69
e
Integrated
Lamprey
Zebrafish
f
NE=0.76
g
Matched types between lamprey and mouse
common (4)
RPCA specific (15)
CCA specific (5)
Matched types between lamprey and chicken
common (1)
CCA specific (0)
RPCA specific (13)
Matched types between lamprey and zebrafish
common (1)
CCA specific (0)
RPCA specific (9)
h
Species Integration
NE
0.39
0.53
0.46
0.69
0.56
0.76
lamprey−mouse
lamprey−chicken
lamprey−zebrafish
Methods
RPCA
CCA

Supplementary Figure 8. Cross-species integration of RGC types.
(a) Transcriptomic integration of lamprey and mouse RGC types via CCA, with both integrated and species-specific clusters presented in separated UMAP plots.
(b) Confusion matrix of the correspondence of mouse RGC types to lamprey ones. Lamprey RGC types were used as a training dataset. NE, Normalized Entropy. See Source Data.
(c)Transcriptomic integration of lamprey and chicken RGC types via CCA, with both integrated and species-specific clusters presented in separated UMAP plots.
(d) Confusion matrix of the correspondence of chicken RGC types to lamprey ones. Lamprey RGC types were used as a training dataset. See Source Data.
(e)Transcriptomic integration of lamprey and zebrafish RGC types via CCA, with both integrated and species-specific clusters presented in separated UMAP plots.
(f) Confusion matrix of the correspondence of zebrafish RGC types to lamprey ones. Lamprey RGC types were used as a training dataset. See Source Data.
(g) Pie charts showing the comparison of matched cell type numbers between CCA and RPCA methods. See Source Data.
(h) Bar plot showing the comparison of NE values between CCA and RPCA. A lower NE value indicates higher prediction confidence. See Source Data.

Figure S9

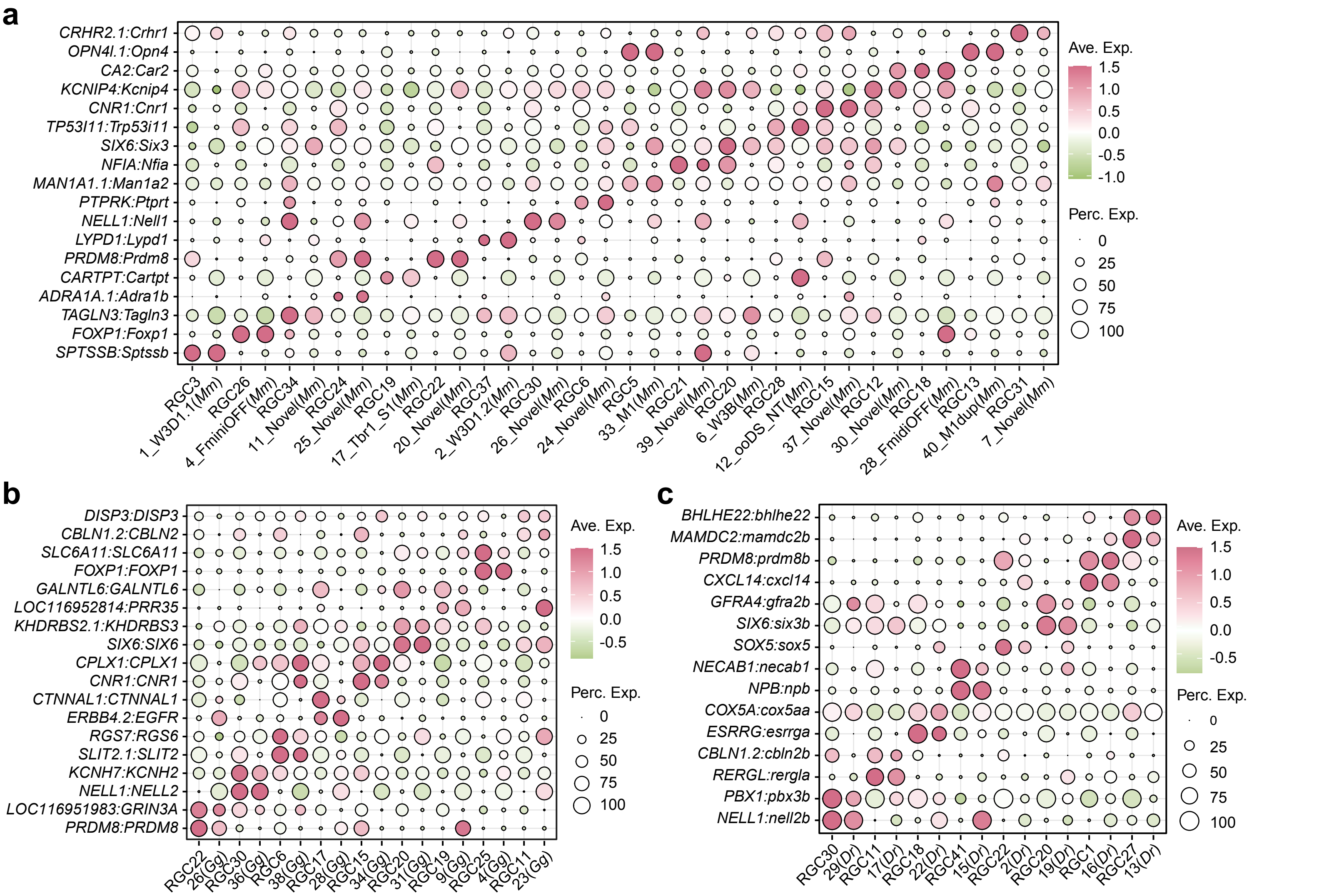
a
CRHR2.1:Crhr1
OPN4l.1:Opn4
CA2:Car2
KCNIP4:Kcnip4
CNR1:Cnr1
TP53I11:Trp53i11
SIX6:Six3
NFIA:Nfia
MAN1A1.1:Man1a2
PTPRK:Ptprt
NELL1:Nell1
LYPD1:Lypd1
PRDM8:Prdm8
CARTPT:Cartpt
ADRA1A.1:Adra1b
TAGLN3:Tagln3
FOXP1:Foxp1
SPTSSB:Sptssb
RGC3
1_W3D1.1(Mm)
RGC26
4_FminiOFF(Mm)
RGC34
11_Novel(Mm)
RGC24
25_Novel(Mm)
RGC19
17_Tbr1_S1(Mm)
RGC22
20_Novel(Mm)
RGC37
2_W3D1.2(Mm)
RGC30
26_Novel(Mm)
RGC6
24_Novel(Mm)
RGC5
33_M1(Mm)
RGC21
39_Novel(Mm)
RGC20
6_W3B(Mm)
RGC28
12_ooDS_NT(Mm)
RGC15
37_Novel(Mm)
RGC12
30_Novel(Mm)
RGC18
28_FmidiOFF(Mm)
RGC13
40_M1dup(Mm)
RGC31
7_Novel(Mm)
Ave. Exp.
1.5
1.0
0.5
0.0
-0.5
-1.0
Perc. Exp.
0
25
50
75
100
b
DISP3:DISP3
CBLN1.2:CBLN2
SLC6A11:SLC6A11
FOXP1:FOXP1
GALNTL6:GALNTL6
LOC116952814:PRR35
KHDRBS2.1:KHDRBS3
SIX6:SIX6
CPLX1:CPLX1
CNR1:CNR1
CTNNAL1:CTNNAL1
ERBB4.2:EGFR
RGS7:RGS6
SLIT2.1:SLIT2
KCNH7:KCNH2
NELL1:NELL2
LOC116951983:GRIN3A
PRDM8:PRDM8
RGC22
26(Gg)
RGC30
36(Gg)
RGC6
38(Gg)
RGC17
28(Gg)
RGC15
34(Gg)
RGC20
31(Gg)
RGC19
9(Gg)
RGC25
4(Gg)
RGC11
23(Gg)
Ave. Exp.
1.5
1.0
0.5
0.0
-0.5
Perc. Exp.
0
25
50
75
100
c
BHLHE22:bhlhe22
MAMDC2:mamdc2b
PRDM8:prdm8b
CXCL14:cxcl14
GFRA4:gfra2b
SIX6:six3b
SOX5:sox5
NECAB1:necab1
NPB:npb
COX5A:cox5aa
ESRRG:esrrga
CBLN1.2:cbln2b
RERGL:rergla
PBX1:pbx3b
NELL1:nell2b
RGC30
29(Dr)
RGC11
17(Dr)
RGC18
22(Dr)
RGC41
15(Dr)
RGC22
2(Dr)
RGC20
19(Dr)
RGC1
16(Dr)
RGC27
13(Dr)
Ave. Exp.
1.5
1.0
0.5
0.0
-0.5
Perc. Exp.
0
25
50
75
100

Supplementary Figure 9. Conserved markers showed conserved RGC types between species.
(a) Dot plot showing conserved markers between matched lamprey and mouse RGC types.
(b) Dot plot showing conserved markers between matched lamprey and chicken RGC types.
(c) Dot plot showing conserved markers between matched lamprey and zebrafish RGC types.
See Source Data.

Figure S10

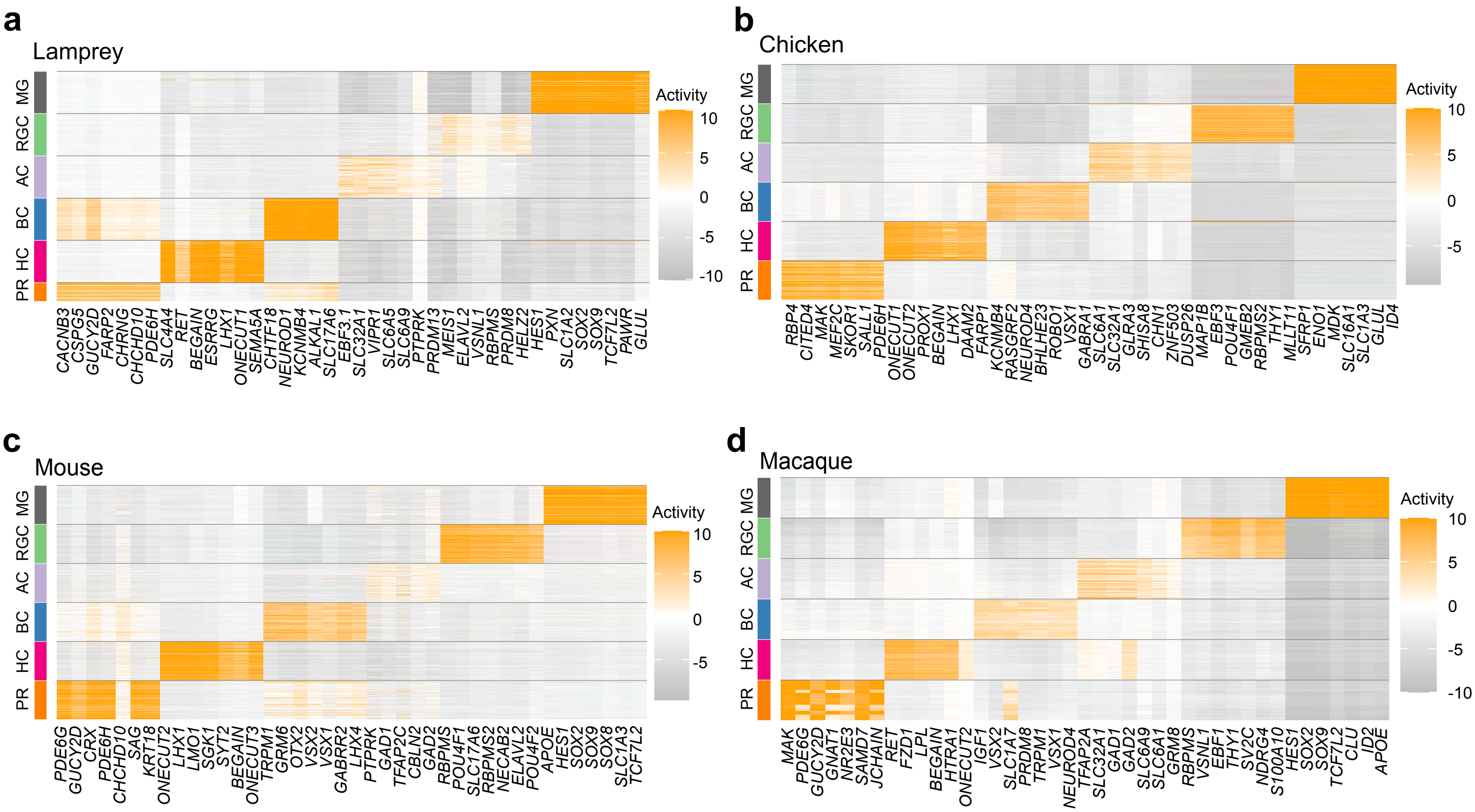
a
Lamprey
MG
RGC
AC
BC
HC
PR
Activity
10
5
0
-5
-10
CACNB3 CSPG5 GUCY2D FARP2 CHRNG CHCHD10 PDE6H SLC4A4 RET BEGAIN ESRRG LHX1 ONECUT1 SEMA5A CHTF18 NEUROD1 KCNMB4 ALKAL1 SLC17A6 EBF3.1 SLC32A1 VIPR1 SLC6A5 SLC6A9 PTPRK PRDM13 MEIS1 ELAVL2 VSNL1 RBPMS PRDM8 HELZ2 HES1 PXN SLC1A2 SOX2 SOX9 TCF7L2 PAWR GLUL
b
Chicken
Activity
RBP4 CITED4 MAK MEF2C SKOR1 SALL1 PDE6H ONECUT1 ONECUT2 PROX1 BEGAIN LHX1 DAAM2 FARP1 KCNMB4 RASGRF2 NEUROD4 BHLHE23 ROBO1 VSX1 GABRA1 SLC6A1 SLC32A1 GLRA3 SHISA8 CHN1 ZNF503 DUSP26 MAP1B EBF3 POU4F1 GMEB2 RBPMS2 THY1 MLLT11 SFRP1 ENO1 MDK SLC16A1 SLC1A3 GLUL ID4
c
Mouse
Activity
PDE6G GUCY2D CRX PDE6H CHCHD10 SAG KRT18 ONECUT2 LHX1 LMO1 SGK1 SYT2 BEGAIN ONECUT3 TRPM1 GRM6 OTX2 VSX2 VSX1 GABRR2 LHX4 PTPRK GAD1 TFAP2C CBLN2 GAD2 RBPMS POU4F1 SLC17A6 RBPMS2 NECAB2 ELAVL2 POU4F2 APOE HES1 SOX2 SOX9 SOX8 SLC1A3 TCF7L2
d
Macaque
Activity
MAK PDE6G GUCY2D GNAT1 NR2E3 SAMD7 JCHAIN RET FZD1 LPL BEGAIN HTRA1 ONECUT2 IGF1 VSX2 SLC1A7 PRDM8 TRPM1 VSX1 NEUROD4 TFAP2A SLC32A1 GAD1 GAD2 SLC6A9 SLC6A1 GRM8 RBPMS VSNL1 EBF1 THY1 SV2C NDRG4 S100A10 HES1 SOX2 SOX9 TCF7L2 CLU ID2 APOE

Supplementary Figure 10. Identification of class-specific regulators in four vertebrate species.
(a) Heatmap showing activities of class-specific regulators in lamprey.
(b) Heatmap showing activities of class-specific regulators in chicken.
(c) Heatmap showing activities of class-specific regulators in mouse.
(d) Heatmap showing activities of class-specific regulators in macaque.

Figure S11

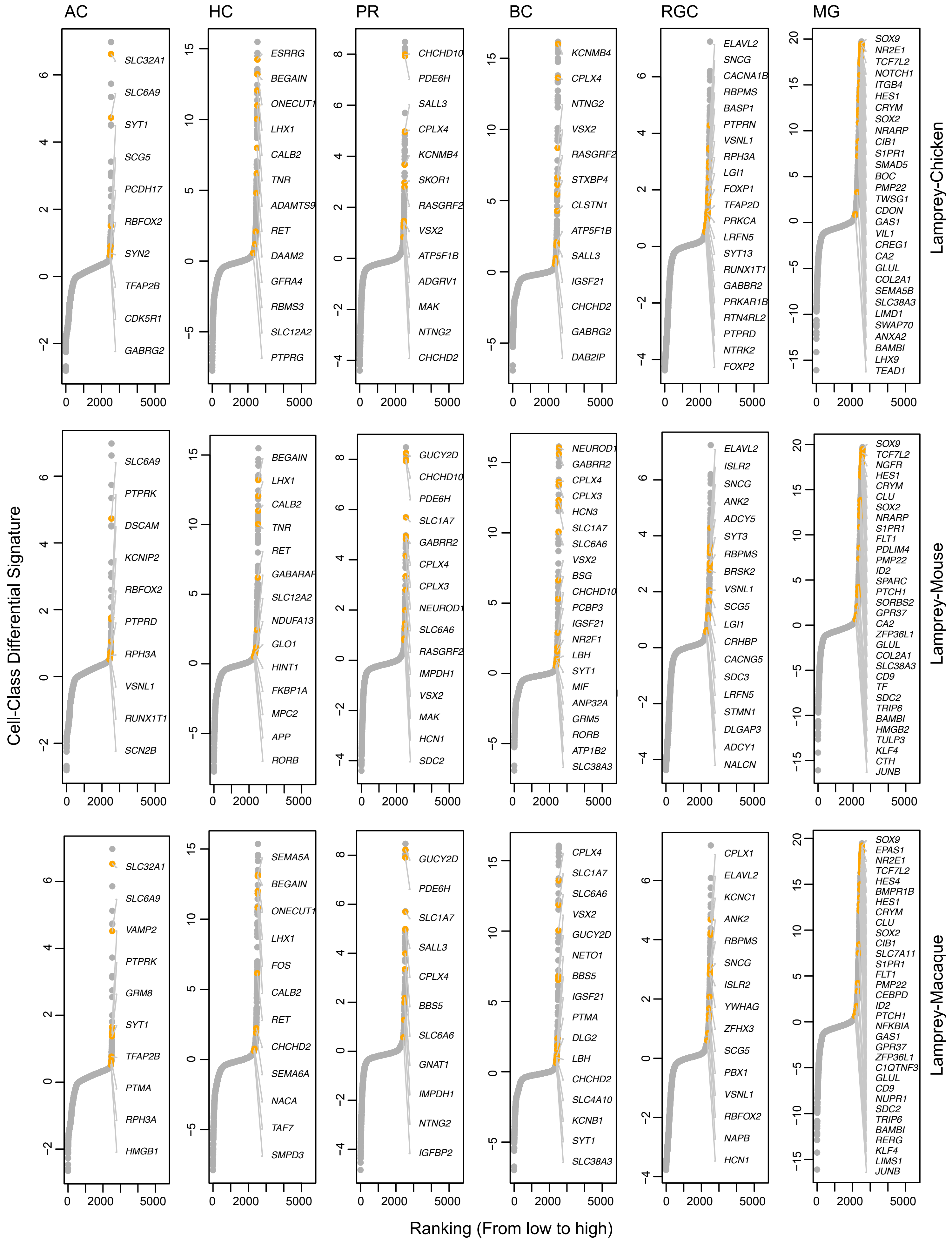
AC
HC
PR
BC
RGC
MG
Lamprey-Chicken
Lamprey-Mouse
Lamprey-Macaque
Cell-Class Differential Signature
Ranking (From low to high)
SLC32A1
SLC6A9
SYT1
SCG5
PCDH17
RBFOX2
SYN2
TFAP2B
CDK5R1
GABRG2
ESRRG
BEGAIN
ONECUT1
LHX1
CALB2
TNR
ADAMTS9
RET
DAAM2
GFRA4
RBMS3
SLC12A2
PTPRG
CHCHD10
PDE6H
SALL3
CPLX4
KCNMB4
SKOR1
RASGRF2
VSX2
ATP5F1B
ADGRV1
MAK
NTNG2
CHCHD2
KCNMB4
CPLX4
NTNG2
VSX2
RASGRF2
STXBP4
CLSTN1
ATP5F1B
SALL3
IGSF21
CHCHD2
GABRG2
DAB2IP
ELAVL2
SNCG
CACNA1B
RBPMS
BASP1
PTPRN
VSNL1
RPH3A
LGI1
FOXP1
TFAP2D
PRKCA
LRFN5
SYT13
RUNX1T1
GABBR2
PRKAR1B
RTN4RL2
PTPRD
NTRK2
FOXP2
SOX9
NR2E1
TCF7L2
NOTCH1
ITGB4
HES1
CRYM
SOX2
NRARP
CIB1
S1PR1
SMAD5
BOC
PMP22
TWSG1
CDON
GAS1
VIL1
CREG1
CA2
GLUL
COL2A1
SEMA5B
SLC38A3
LIMD1
SWAP70
ANXA2
BAMBI
LHX9
TEAD1

Supplementary Figure 11. Comparison of class-specific regulators between the lamprey and three jawed species.
Waterfall plots depicting all inferred proteins in the lamprey, ranked by their cell-class specificity. Top active regulators in the lamprey that are also activated in chicken (top), mouse (middle), and macaque (bottom) are highlighted in yellow and shown with the gene name.

Supplementary Table 1. Published Datasets Used in This Study

Dataset	GEO	Dataset URL	Usage	Reference
Mouse PR	GSE135406	https://github.com/jiewwwang/Single-cell-retinal-regeneration	Cross-Species Integration	Hoang et al. (*Ref 31*)
Mouse BC	GSE81905	https://singlecell.broadinstitute.org/single_cell/study/SCP3/retinal-bipolar-neuron-drop-seq	Cross-Species Integration	Shekhar et al. (*Ref 33*)
Chicken BC	GSE159107	https://singlecell.broadinstitute.org/single_cell/study/SCP1159/a-cell-atlas-of-the-chick-retina-based-on-single-cell-transcriptomics	Cross-Species Integration	Yamagata et al. (*Ref 19*)
Zebrafish BC	GSE237214	https://www.ncbi.nlm.nih.gov/geo/query/acc.cgi?acc=GSE237214	Cross-Species Integration	Hellevik et al. (*Ref 35*)
Chicken HC	GSE159107	https://singlecell.broadinstitute.org/single_cell/study/SCP1159/a-cell-atlas-of-the-chick-retina-based-on-single-cell-transcriptomics	Cross-Species Integration	Yamagata et al. (*Ref 19*)
Mouse SAC	GSE132555	https://www.ncbi.nlm.nih.gov/geo/query/acc.cgi?acc=GSM3872341	Cross-Species Integration	Peng et al. (*Ref 30*)
Mouse AC	GSE149715	https://www.ncbi.nlm.nih.gov/geo/query/acc.cgi?acc=GSE149715	Cross-Species Integration	Yan et al. (*Ref 34*)
Chicken AC	GSE159107	https://singlecell.broadinstitute.org/single_cell/study/SCP1159/a-cell-atlas-of-the-chick-retina-based-on-single-cell-transcriptomics	Cross-Species Integration	Yamagata et al. (*Ref 19*)
Mouse RGC	GSE137400	https://singlecell.broadinstitute.org/single_cell/study/SCP509/mouse-retinal-ganglion-cell-adult-atlas-and-optic-nerve-crush-time-series	Cross-Species Integration	Tran et al. (*Ref 52*)

Chicken RGC	GSE159107	https://singlecell.broadinstitute.org/single_cell/study/SCP1159/a-cell-atlas-of-the-chick-retina-based-on-single-cell-transcriptomics	Cross-Species Integration	Yamagata et al. (*Ref 19*)
Zebrafish RGC	GSE152842	https://drive.google.com/drive/folders/1baRKtDkD4d8-6tG8P9v8VcjtUDpWeq5m	Cross-Species Integration	Kolsch et al. (*Ref 74*)
Mouse	GSE149715	https://www.ncbi.nlm.nih.gov/geo/query/acc.cgi?acc=GSE149715	Protein Activity Analysis	Yan et al. (*Ref 34*)
Chicken	GSE159107	https://singlecell.broadinstitute.org/single_cell/study/SCP1159/a-cell-atlas-of-the-chick-retina-based-on-single-cell-transcriptomics	Protein Activity Analysis	Yamagata et al. (*Ref 19*)
Macaque	GSE118480	https://www.ncbi.nlm.nih.gov/geo/query/acc.cgi?acc=GSE118480	Protein Activity Analysis	Peng et al. (*Ref 30*)

The Dataset URL links to the processed dataset if it is used.

For the raw dataset, we preprocessed the data following the Seurat guided clustering tutorial (https://satijalab.org/seurat/articles/pbmc3k_tutorial).

Supplementary Table 2: The Information of RNA Probes

Target Genes	Primers (The T7 promoter sequences are placed in the reverse primer and indicated with lower cases)	Probe Length (bp)	Tag	Sequence Reference
NEUROD1	Forward: GGACTGCGAATCTCAACACG; reverse: gaaattaatacgactcactatagggCCGCGTAGTGAAACCGATAG	1037	DIG	XM_032946356.1
SLC32A1	Forward: CAACTTCCTCGTCATCGGCTA; reverse: gaaattaatacgactcactatagggACAGAGGCTGCATTGGAACAA	1074	DIG	XM_032952818
GLUL	Forward: GTTCGGGAAGCACTCTCTCC; reverse: gaaattaatacgactcactatagggGCGGAGAACTCGTGTATGCT	1020	DIG	XM_032973232.1
RHO	Forward: TGAACGGCACAGAGGGAGAA; reverse: gaaattaatacgactcactatagggGAGGCTCCCGAGTCTTCATC	1006	DIG<br>Flu	XM_032971488.1
Red-opsin	Forward: GGCAGGGGGCGATGTTC; reverse: gaaattaatacgactcactatagggCGTCGTCCACTTTCTTCCCA	1014	DIG<br>Flu	XM_032971650.1
GNAT1	Forward: AACGTCAAGTTCGTGTTCGAC; reverse: gaaattaatacgactcactatagggCCCATCAGTTTCCCCAAACAC	1018	DIG<br>Flu	XM_032954230
GNAT2	Forward: AGAAGAAGCTCGCCGAAGATG; reverse: gaaattaatacgactcactatagggAGAGGCCGCAGTCTTTGAGG	1002	DIG<br>Flu	XM_032971636
SLC17A6	Forward: GCGAGCTGACTGCAATGTTT; reverse: gaaattaatacgactcactatagggAGCACGTACCAACAGGACAG	1041	DIG	XM_032960051.1
OPN4l.1	Forward: ATGGTCTCCCTCTCGCAGAT; reverse: gaaattaatacgactcactatagggAGGATGGACTCCTCTCGTCC	1126	DIG	XM_032944209.1
CHAT	Forward: TCCCCCACTTCGAGTGTAGT; reverse: gaaattaatacgactcactatagggTAAACCCCAAGCAATGCCCA	1041	DIG	XM_032959092.1
MEGF10	Forward: TTCTGTGAGGAGGTTTGCCC; reverse: gaaattaatacgactcactatagggTTCTCTGGCATGTGGTTCCC	1183	DIG	XM_032953473.1

DIG: Digoxigenin; Flu: Fluorescein

Supplementary Table 3. Parameters for Removing Low-quality Cells in Lamprey Cell Classes.

Class	nFeature_RNA	nCount_RNA
AC	>700 & <5000	>950 & <15000
BC	>300 & <7000	>500 & <32000
HC	>1500 & <7200	>2500 & <40000
MG	>330 & <9000	>500 & <61500
PR	>350 & <4350	>500 & <14800
RGC	>1000 & <6000	>1400 & <29500

Supplementary Table 4. Cross-species Integration Parameters and Hyperparameters

Integration	Integration method	Down-sample Parameter (# cells per type)	k. anchor
Lamprey-mouse PR (Fig. 2d)	CCA	NA	Default
Lamprey-mouse BC (Fig. 3f)	CCA	NA	Default
Lamprey-mouse BC (Supplementary Fig. 4g)	RPCA	Lamprey 50; Mouse nonRBC 300, RBC 800	10
Lamprey-mouse BC (Fig. 3h)	RPCA	Lamprey 50; Mouse nonRBC 300, RBC 1000	20
Lamprey-chicken BC (Fig. 3h)	RPCA	Lamprey 50; Chicken 200	20
Lamprey-zebrafish BC (Fig. 3h)	RPCA	Lamprey 300*; Zebrafish 200	20
Mouse-chicken BC (Supplementary Fig. 5d)	RPCA	Mouse NA; Chicken 200	20
Mouse-zebrafish BC (Supplementary Fig. 5a)	RPCA	Lamprey 300; Zebrafish 300	20
Lamprey-chicken HC (Fig. 4d)	CCA	NA	Default
Lamprey-mouse SAC (Fig. 4j)	CCA	NA	Default
Lamprey-mouse AC (Fig. 5a)	CCA	Lamprey NA; Mouse 200	Default
Lamprey-chicken AC (Fig. 5b)	CCA	Lamprey 100; Chicken 100	Default
Lamprey-mouse RGC (Fig. 6a)	RPCA	Lamprey 200; Mouse 200	20
Lamprey-chicken RGC (Fig. 6a)	RPCA	Lamprey 200; Chicken 200	20
Lamprey-zebrafish RGC (Fig. 6a)	RPCA	Lamprey 200; Zebrafish 200	20
Lamprey-mouse RGC (Supplementary Fig. 8a)	CCA	Lamprey 100; Mouse 100	Default
Lamprey-chicken RGC (Supplementary Fig. 8c)	CCA	Lamprey 200; Chicken 200	Default
Lamprey-zebrafish RGC (Supplementary Fig. 8e)	CCA	Lamprey 200; Zebrafish 200	Default

NA, Not Applicable. All the cells were used.

* The number of cells per subclass.

Supplementary Table 5. Downsampling Parameters for Generating the ARACNe-AP Network.

Species	Down-sampled Cell Number per Class per Sample	Number of Sample
Lamprey	NonRGC 200; RGC 400	2
Chicken	150	1
Mouse	100	1
Macaque	200	3
