# Supplementary material for: Molecular characterization of the sea lamprey retina illuminates the evolutionary origin of retinal cell types
Source: Nat Commun. 2024 Dec 30;15:10761. doi: 10.1038/s41467-024-55019-x (PMC11685597; doi:10.1038/s41467-024-55019-x)
Supplement: Supplementary file 2 — Description of Additional Supplementary Files [file 41467_2024_55019_MOESM2_ESM.docx]

**Description of Additional Supplementary Files**

**Supplementary Data 1**: Correspondence of Gene Names Across GTF Files and Count Matrix.

**Supplementary Data 2**: Orthologous Gene Relationships Across Lamprey, Zebrafish, Chicken, Mouse, Macaque, and Human.

2a) Orthologous Genes Across Lamprey, Zebrafish, Chicken, Mouse, Macaque, and Human.

2b) Orthologous Genes between Lamprey and Zebrafish.

2c) Orthologous Genes between Lamprey and Chicken.

2d) Orthologous Genes between Lamprey and Mouse.

2e) Orthologous Genes between Lamprey and Mouse.

2f) Orthologous Genes between Lamprey and Human.
