# Supplementary material for: Molecular characterization of the sea lamprey retina illuminates the evolutionary origin of retinal cell types
Source: Nat Commun. 2024 Dec 30;15:10761. doi: 10.1038/s41467-024-55019-x (PMC11685597; doi:10.1038/s41467-024-55019-x)
Supplement: Supplementary file 5 — Reporting Summary [file 41467_2024_55019_MOESM5_ESM.pdf]

nature portfolio

Corresponding author(s): Yi-Rong Peng

Last updated by author(s): Nov 6, 2024

Reporting Summary

Nature Portfolio wishes to improve the reproducibility of the work that we publish. This form provides structure for consistency and transparency in reporting. For further information on Nature Portfolio policies, see our Editorial Policies and the Editorial Policy Checklist.

Statistics

For all statistical analyses, confirm that the following items are present in the figure legend, table legend, main text, or Methods section.

n/a	Confirmed	
☐	☒	The exact sample size (*n*) for each experimental group/condition, given as a discrete number and unit of measurement
☐	☒	A statement on whether measurements were taken from distinct samples or whether the same sample was measured repeatedly
☐	☒	The statistical test(s) used AND whether they are one- or two-sided<br>*Only common tests should be described solely by name; describe more complex techniques in the Methods section.*
☐	☒	A description of all covariates tested
☐	☒	A description of any assumptions or corrections, such as tests of normality and adjustment for multiple comparisons
☐	☒	A full description of the statistical parameters including central tendency (e.g. means) or other basic estimates (e.g. regression coefficient) AND variation (e.g. standard deviation) or associated estimates of uncertainty (e.g. confidence intervals)
☐	☒	For null hypothesis testing, the test statistic (e.g. *F*, *t*, *r*) with confidence intervals, effect sizes, degrees of freedom and *P* value noted<br>*Give P values as exact values whenever suitable.*
☒	☐	For Bayesian analysis, information on the choice of priors and Markov chain Monte Carlo settings
☒	☐	For hierarchical and complex designs, identification of the appropriate level for tests and full reporting of outcomes
☐	☒	Estimates of effect sizes (e.g. Cohen's *d*, Pearson's *r*), indicating how they were calculated

Our web collection on statistics for biologists contains articles on many of the points above.

Software and code

Policy information about availability of computer code

Data collection	Libraries of single-cell and single nuclei RNAseq were sequenced on the Illumina NovaSeq S4 platform. TruSeq libraries were sequenced on the Illumina NextSeq 500 platform.
Data analysis	Tru-Seq data were analyzed with SAMtools (v1.9), StringTie2 (v2.1.4), and HiSat2 (v2.2.0). ScRNA-seq data were analyzed with Cell Ranger (10X Genomics), Seurat R package (v4.3.0), Harmony (v0.1.1), singleR(v2.2.0), XGBoost (v1.7.3.1), OrthoFinder (v2.4.0), ARACNe-AP, and VIPER (v1.34.0). Customized analysis tools were deposited to https://github.com/PengYRLab/LampreyRetinalCellAtlas.

For manuscripts utilizing custom algorithms or software that are central to the research but not yet described in published literature, software must be made available to editors and reviewers. We strongly encourage code deposition in a community repository (e.g. GitHub). See the Nature Portfolio guidelines for submitting code & software for further information.

Data

Policy information about availability of data

All manuscripts must include a data availability statement. This statement should provide the following information, where applicable:

- Accession codes, unique identifiers, or web links for publicly available datasets
- A description of any restrictions on data availability
- For clinical datasets or third party data, please ensure that the statement adheres to our policy

The raw TruSeq data have been deposited to the Sequence Read Archive (SRA) with accession numbers SRX26477354 and SRX26477355. The scRNA-Seq data

generated in the study have been deposited to the Gene Expression Omnibus (GEO) under accession ID GSE236005. Previously published data utilized in this paper were downloaded from GEO repositories with the following accession numbers: GSE135406 (mouse PR), GSE81905 (mouse BC), GSE159107 (chicken), GSE237214 (zebrafish BC), GSE132555 (mouse SAC), GSE149715 (mouse AC), GSE137400 (mouse RGC), GSE152842 (zebrafish RGC), GSE118480 (macaque). This information is also summarized in Supplementary Table 1. The genome annotation files have been deposited to Zenodo (https://doi.org/10.5281/zenodo.13975013). Fluorescence in situ images and immunohistological images have been deposited to Zenodo (https://doi.org/10.5281/zenodo.13988889).

Research involving human participants, their data, or biological material

Policy information about studies with human participants or human data. See also policy information about sex, gender (identity/presentation), and sexual orientation and race, ethnicity and racism.

Reporting on sex and gender	NA
Reporting on race, ethnicity, or other socially relevant groupings	NA
Population characteristics	NA
Recruitment	NA
Ethics oversight	NA

Note that full information on the approval of the study protocol must also be provided in the manuscript.

Field-specific reporting

Please select the one below that is the best fit for your research. If you are not sure, read the appropriate sections before making your selection.

☒ Life sciences ☐ Behavioural & social sciences ☐ Ecological, evolutionary & environmental sciences

For a reference copy of the document with all sections, see nature.com/documents/nr-reporting-summary-flat.pdf

Life sciences study design

All studies must disclose on these points even when the disclosure is negative.

Sample size	To capture all the cell types in the retina, a total of 21,474 cells were included in this study
Data exclusions	Data was filtered out based on a minimum of 400 expressed genes per cell and a minimum of 10 cells with detected expression per gene, and a high percentage of mitochondrial and ribosomal gene expression.
Replication	Retinal cells from at least two animals were used for this study.
Randomization	All the sequencing data were mixed from different animal and batches.
Blinding	The investigators were blind to groups.

Reporting for specific materials, systems and methods

We require information from authors about some types of materials, experimental systems and methods used in many studies. Here, indicate whether each material, system or method listed is relevant to your study. If you are not sure if a list item applies to your research, read the appropriate section before selecting a response.

Materials & experimental systems

n/a	Involved in the study	
☐	☒	Antibodies
☒	☐	Eukaryotic cell lines
☒	☐	Palaeontology and archaeology
☐	☒	Animals and other organisms
☒	☐	Clinical data
☒	☐	Dual use research of concern
☒	☐	Plants

Methods

n/a	Involved in the study	
☒	☐	ChIP-seq
☒	☐	Flow cytometry
☒	☐	MRI-based neuroimaging

Antibodies

Antibodies used	Mouse anti-PKCa (1:2000, Abcam # ab31, MC5) antibody was used. Donkey anti-mouse secondary antibody, Alexa Fluor 488 (Jackson Immunoresearch #715-545-150) was used at 1:1000.
Validation	The primary antibody is a commercial antibody and validated by the company.

Animals and other research organisms

Policy information about studies involving animals; ARRIVE guidelines recommended for reporting animal research, and Sex and Gender in Research

Laboratory animals	NA
Wild animals	Sea lamprey, Petromyzon marinus Linnaeus 1758, were provided by the Hammond Bay Biological Station of the United States Geological Survey (USGS), Millersburg, MI, USA.
Reporting on sex	NA
Field-collected samples	NA
Ethics oversight	Lamprey tissue collection was carried out in accordance with the recommendations of the Guide for the Care and Use of Laboratory Animals of the National Institutes of Health, USA, and was approved by the University of California, Los Angeles, Animal Research Committee.

Note that full information on the approval of the study protocol must also be provided in the manuscript.

Plants

Seed stocks	NA
Novel plant genotypes	NA
Authentication	NA
